# Gaming to see: action video gaming is associated with enhanced processing of masked stimuli

**DOI:** 10.3389/fpsyg.2014.00070

**Published:** 2014-02-05

**Authors:** Carsten Pohl, Wilfried Kunde, Thomas Ganz, Annette Conzelmann, Paul Pauli, Andrea Kiesel

**Affiliations:** ^1^Department of Psychology, Cognitive Psychology, University of WürzburgWürzburg, Germany; ^2^Hospital for Psychiatry, Psychotherapy and Psychosomatic Medicine Lohr am MainLohr am Main, Germany; ^3^University of Applied Sciences EsslingenEsslingen, Germany; ^4^Department of Psychology, Biological Psychology, Clinical Psychology and Psychotherapy, University of WürzburgWürzburg, Germany

**Keywords:** masked priming, action video gaming, unconscious processing, prime visibility, expertise

## Abstract

Recent research revealed that action video game players outperform non-players in a wide range of attentional, perceptual and cognitive tasks. Here we tested if expertise in action video games is related to differences regarding the potential of shortly presented stimuli to bias behavior. In a response priming paradigm, participants classified four animal pictures functioning as targets as being smaller or larger than a reference frame. Before each target, one of the same four animal pictures was presented as a masked prime to influence participants' responses in a congruent or incongruent way. Masked primes induced congruence effects, that is, faster responses for congruent compared to incongruent conditions, indicating processing of hardly visible primes. Results also suggested that action video game players showed a larger congruence effect than non-players for 20 ms primes, whereas there was no group difference for 60 ms primes. In addition, there was a tendency for action video game players to detect masked primes for some prime durations better than non-players. Thus, action video game expertise may be accompanied by faster and more efficient processing of shortly presented visual stimuli.

## Introduction

Over the last three decades, public as well as scientific interest in action video gaming focused mainly on negative consequences such as video game addiction (e.g., Griffiths and Meredith, 2009) or promoting the likelihood of aggressive behavior (e.g., Anderson et al., 2003; Carnagey et al., 2007). While there is still a lively debate whether action video games do actually increase aggressive behavior or whether effects found in the laboratory can be transferred to account for aggressive behavior in real live (e.g., Anderson et al., 2010; Bushman et al., 2010; Ferguson and Kilburn, 2010; Ferguson et al., 2013), research in recent years has also revealed several positive side-effects of playing action video games on vision, perception, attention, and cognitive control.

In a series of studies, Green and Bavelier(2003; 2006a; 2006b; 2007; see Green et al., 2010a, for a review) compared action video game players and novices regarding their performance in many standard paradigms of cognitive psychology, like the flanker task, enumeration task, useful field of view task, attentional blink task, multiple object tracking task, perceptual load paradigm, and crowding paradigm. Compared to novices, action video game players performed better in peripheral and central vision tasks, better under dual task conditions, and action gamers displayed evidence of greater attentional resources, an enhanced spatial distribution of attention, and a greater temporal and spatial resolution of attention. Action video game usage also seems to promote parallel processing, as action video game players were able to enumerate and track substantially more items at once than novices (Green and Bavelier, 2006b; see also Trick et al., 2005). Further, action video game players also showed benefits for multisensory processing when visual and auditory stimuli were presented in close temporal succession (Donohue et al., 2010). Video game experience was also associated with an increased ability to switch between two tasks (Colzato et al., 2010), enhanced monitoring and updating of working memory (Colzato et al., 2013), and improved probabilistic inference (Green et al., 2010b). These tasks are considerably different from the situation of gaming itself which suggests a substantial transfer of training.

Moreover, training studies suggest that differences between gamers and non-gamers are not just correlational, but that there is a causal relationship between action video game play and improved perceptual and cognitive abilities (e.g., Green and Bavelier, 2003, 2006a,b, 2007; Li et al., 2009; Strohbach et al., 2012). Novices who were trained with an action video game (Medal of Honor, Call of Duty 2, or Unreal Tournament) performed better than novices who were trained with a non-action video game (Tetris or The Sims). Ten to 50 h of training with an action game was sufficient to induce considerable impoverishments.

Regarding more basic effects on visual processing, Li et al. (2009) reported a long-lasting enhancement of contrast sensitivity through action video game playing and intensive training. Contrast sensitivity is “the ability to detect small increments in shades of gray on a uniform background” (Li et al., 2009, p. 549). It is seen as “one of the most basic visual functions that commonly deteriorate with aging” (Caplovitz and Kastner, 2009, p. 527) and it is assumed to be important in many different visual tasks. Contrast sensitivity was measured with a detection task for a briefly presented gabor patch. Detection performance was better in action video game players and action video game trained participants than for novices and non-action video game trained participants.

Furthermore, using a lateral masking paradigm, Li et al. (2010) found that action video game training also influences the temporal dynamics of vision. A central gabor patch was presented as target. This gabor patch was masked by two vertically flanking gabor patches. The SOA of the masks was varied in order to create forward or backward masking. Participants had to detect the central gabor patch. Action video game players showed reduced backward masking compared to non-action game players. This pattern of result was replicated in a training study, suggesting a causal relationship between action video gaming and improved cortical dynamics.

The aim of the current study is to further increase our knowledge of types of processes might be affected by video game expertise. Here, we asked whether action video game expertise enhances not just the ability to detect visible stimuli but also the ability to process stimuli that are hardly visible. To assess processing of such stimuli, we used a masked priming paradigm (e.g., Dehaene et al., 1998; Dell'Acqua and Grainger, 1999; Naccache and Dehaene, 2001; Kunde et al., 2003; Kiesel et al., 2006a) and assessed stimulus-response translation processing based on masked prime stimuli. In addition, we also assessed visibility of the masked stimuli in a visibility test.

There are two reasons for this approach. First, using masked stimuli helps to scrutinize the level of neural processing that action video gaming affects. The impact of masked stimuli is based on what Lamme calls the “forward sweep” of stimulus processing (Lamme, 2003, 2006). This relates to fast forward processing of retinal input within the first about 100 ms. Observers are not aware of stimuli at that level of processing, and normally do not become aware of them later, provided the visual representation is destroyed by masking. It is only when the visual percept is stabilized by recurrent neural processing that consciousness kicks in. Demonstrating that video gaming impacts the processing of masked stimuli would thus reveal that this impact occurs already at the first neural processing sweep, rather than at the level of later recurrent processing. A second reason relates to potential strategic influences on performance. Action video gamers might adapt performance according to demand characteristics, particularly so if they assume that game experience was a reason for choosing them as participants (cf. Boot et al., 2011; Kristjánsson, 2013). In other words gamers might make larger efforts for fast responding just because they consider themselves as a specific sample supposed to respond quickly. Whereas it is obviously possible to change responses to stimuli that are consciously discernible, it is far less obvious how to change the impact of prime stimuli that are hardly visible. In fact, with the pictorial stimulus material we used here, subliminal primes affect performance largely independent of response times to conscious targets (except perhaps for very slow responses)—a finding we will replicate here (Kiesel et al., 2009; Heinemann et al., 2010). So while gamers might try to speed up responding due to demand characteristics, this is unlikely to affect the congruency effects exerted by masked primes. Such congruency effects are thus a less demand-contaminated measure of visual processing than RTs to visible stimuli are, although demand effects that do not leave a trace in RT cannot ultimately be ruled out.

**Figure 1:**
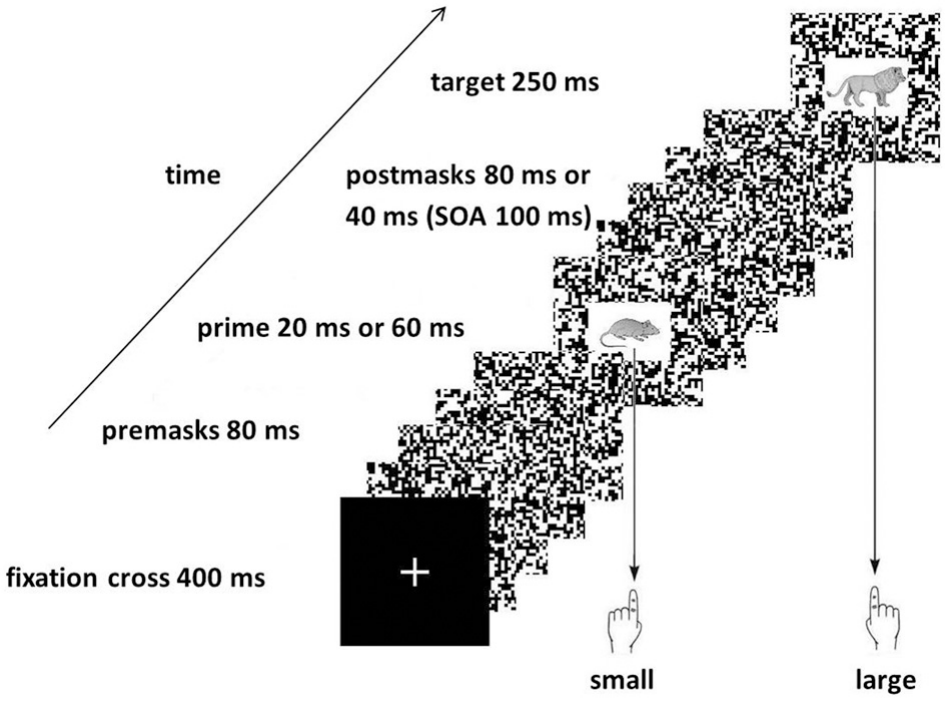
**Sequence of the events in the Experiment.** The Figure shows an incongruent trial because the prime picture (the mouse) would afford the left response indicating “small” while the target picture (the lion) affords the right response indicating “large.”

For this experiment we used a picture priming paradigm we had already established in our laboratory and that produced stable priming effects (Pohl et al., 2010). Participants saw pictures of animals that could be easily classified as being smaller or larger than a reference object. Prior to each target picture, one of the same four animal pictures is presented as prime. If this prime suggests the same response as the target (congruent), participants usually respond faster and less error-prone compared to when prime and target suggest different responses (incongruent). To reduce visibility of the prime stimuli, primes are presented very short and additionally they are masked by random dot masks (see Figure 1). Here in this study, we presented primes either for 20 or 60 ms to assess stimulus-response translation processes of hardly visible and more visible stimuli. If video game players (VGP) process hardly visible stimuli more efficiently than non-video game players (NVGP), we expect larger congruence effects for the players.

This design differs substantially from the lateral priming design Li et al. (2010) used. In their study, the correct temporal detection of a gabor patch was assessed. Participants had to decide in which of two intervals the gabor patch was presented. In contrast, in the present study, we were interested to find out whether action video gaming is associated with enhanced processing of masked primes in a way that affords processing of hardly visible stimuli according to their identity. In order to perform the task, participants had to identify the pictured animals and they were asked to categorize the pictured animals according their size in real life. Masked prime processing occurs when participants apply the task instructions already on the masked primes (cf. Dehaene et al., 1998). That would indicate that also masked animal pictures were processed in the same way as the clearly visible targets, i.e., they are identified and categorized according to their size in real life.

In addition, participants performed a prime visibility test after the priming study. Here, primes were presented 20, 40, 60, 80, or 100 ms. This visibility test served two purposes. First, we wanted to check to which degree participants could see the primes. Second, we aimed to replicate the findings of Li et al. (2009) as well as Li et al. (2010) and expected that VGPs can identify the primes more often correctly than NVGPs.

## Material and methods

### Participants

To recruit participants we placed two advertisements on a regional online job platform, one for people who play action video games (such as Call of Duty: Modern Warfare 3, Battlefield 3, Counter Strike, Boarderlands, or Medal of Honor) and one for people who play no computer games. Then we used the platform www.SoSciSurvey.de to identify a sufficient number of VGPs and NVGPs. The criteria for VGPs were that they indicated playing at least 8 to 10 h per week during the last year. NVGPs were persons who reported that they currently played no computer games and seldom did so in the past. Consequently, we conjecture that participants were aware that they were chosen for the experiment either because of their gaming experience or because of the lack of it.

We a priori decided to match the VGP and NVPG groups for age, gender, and IQ in order to guarantee that general cognitive abilities were equally distributed in both groups and both groups were consistent in terms of demographic makeup. Accordingly, from the 360 persons who participated in the online survey, we selected a sample of sixty healthy male adults between 18 and 29 years (with an average age of 23.7 years) who met either the criteria for VGPs (30 participants) or NVGPs (30 participants). They gave informed consent to participate in a study to investigate emotional, mental and behavioral processes. Data of five participants per group were not included in the analyses because afterwards they admitted that they did not fulfill the criteria for VGP (3 participants), declared to have a mental illness (1), or reported to have not followed the instruction in the prime visibility task correctly (4). Further, data of two participants of the NVGP group^1^ were discarded to ensure that the two groups did not differ with respect to intelligence. All participants reported having normal or corrected-to-normal vision, and were not familiar with the purpose of the experiment.

To check whether the VGP and the NVGP groups are similar expect for the gaming experience, we assessed fluid intelligence (via the SPM) as well as age. Intelligence was measured with a pen and pencil version of the Raven Standard Progressive Matrices Test (SPM; Heller et al., 1998) which was given without time limit. This test for fluid intelligence is widely used in research as well as in practice (Raven, 2000) for the measurement of general intelligence (reasoning ability of Spearman's g factor) and does not depend on language. For the analysis of fluid intelligence we calculated with the raw scores of the SPM because of missing current norms for the German sample (Heller et al., 1998). Independent samples *t*-tests showed no significant group differences for fluid intelligence, *t*_(48)_ = 1.67, *p* > 0.10 and age, *t*_(48)_ = −1.30, *p* = 0.20 (see Table 1 for mean values).

**Table 1:** **Mean values for demographic characteristics (standard deviations are given in brackets), performance for congruent and incongruent primes that were presented 20 and 60 ms, separately for VGPs and NVGPs (standard errors are given in brackets)**.

	**VGPs**		**NVGPs**	
SPM raw score	55.6 (3.2)		54.0 (3.5)	
Age	23.0 (3.0)		24.0 (2.8)	
**Congruence**	**Incongruent**	**Congruent**	**Incongruent**	**Congruent**
**20 ms PRIMES**				
RT (ms)	397 (7.6)	381 (9.2)	423 (7.6)	417 (9.2)
PE (%)	4.4 (0.8)	3.2 (0.7)	3.5 (0.8)	2.3 (0.7)
**60 ms PRIMES**				
RT (ms)	432 (8.7)	376 (8.8)	462 (8.7)	405 (8.8)
PE (%)	11.3 (1.8)	2.4 (0.5)	9.2 (1.8)	2.6 (0.5)

### Apparatus and stimuli

The experiment took place in a dimly lit room. An IBM compatible computer with a 17 inch VGA-Display and the software package E-Prime™ (Schneider et al., 2002) were used for stimulus presentation and response sampling. Stimulus presentation was synchronized with the vertical retraces of a 100-Hz monitor, resulting in a refresh rate of 10 ms. Responses were executed with the index fingers of both hands and collected with an external keyboard with three response keys (1.7 cm width, distance 0.2 cm); the middle response key was not used.

The pictures used as targets and primes were derived from a set of gray scale shaded images of “Snodgrass and Vanderwart-like” objects (Rossion and Pourtois, 2004; see http://wiki.cnbc.cmu.edu/Objects)^2^. The target set consisted of four animal pictures (mouse, snail, lion and zebra,) that could be easily classified as being smaller or larger than a frame measuring 40 × 40 cm (mouse and snail—smaller; lion and zebra—larger). The same four pictures were used as primes. To vary visibility of the primes, primes were either presented for 20 ms or for 60 ms. All pictures were drawn in a white rectangle extending 2 cm high by 3 cm wide. Masks were random dot patterns extending 7.5 × 7.5 cm. They were constructed such that always 4 × 4 pixels were chosen randomly to be white or black. To increase masking, we presented always four different random dot patterns with a total duration of 80 ms as premask and four or two different random dot patterns with a total duration of 80 or 40 ms, respectively. Additionally, the prime as well as the target picture were also presented on a random dot pattern background (see Figure 1).

### Procedure and design

The experiment was completed in a single session that lasted approximately 90 min and that was compensated with 12 Euro. The session consisted of two parts. First, participants executed a priming experiment. Second, a prime discrimination task was administered. Afterwards, individual intelligence was measured with the SPM.

In the following the two parts of the computer experiment are described in more detail.

### Priming experiment

The sequence of the events in a trial in the priming experiment is shown in Figure 1. On each trial, a fixation cross was presented for 400 ms. Then the premasks were presented for 80 ms followed by the prime presented either for 20 or 60 ms. Then the postmasks were presented either for 80 or 40 ms, respectively, so that the Simulus Onset Asynchrony (SOA) between prime and target presentation was always 100 ms. Finally, the target was presented directly after the postmasks for 250 ms. After response execution a fixed time interval of 1000 ms elapsed before the next trial started.

Participants were instructed to categorize the depicted animals as being smaller or larger than a reference frame (40 × 40 cm) and to respond as fast as possible while they should avoid to make errors. Participants had to press a left key with the left index finger to indicate “smaller” and a right key with the right index finger to indicate “larger” as fast and as accurately as possible. Errors were indicated by the German word for wrong (“Falsch!”) presented in red in the lower part of the monitor. Response times were recorded from the onset of the target until the onset of the response.

There were 32 (4 × 4 × 2) different combinations of target, prime and prime duration (20 or 60 ms) that were presented 10 times each. After each block of 64 trials, participants were allowed a short, self-paced break. In addition, mean response times and percentage of errors were fed back to encourage participants to increase their performance.

### Assessment of prime visibility

After the priming experiment, we tested prime visibility with a separate prime discrimination task. In general, the stimuli as well as their sequence was comparable to the priming experiment. However, in this experimental part, participants were fully informed about the precise structure of a trial and the presence of the masked primes. This time, participants were asked to discriminate whether the prime picture was smaller or larger than the reference object. For the discrimination task, participants were instructed to take their time and to try to be as accurate as possible. In order to avoid that unconsciousness congruence effects influence the free response choice (see Schlaghecken and Eimer, 2004; Kiesel et al., 2006b), there was an interval of 800 ms after target offset, in which no response was possible (e.g., Vorberg et al., 2003).

In order to get a more graded assessment of prime visibility, we varied prime duration from 20 to 100 ms in steps of 20 ms. Thus, in a trial the prime picture was presented either for 20, 40, 60, 80 or 100 ms. As in the priming part of the experiment, the SOA was held constant for 100 ms. Therefore, postmasks were presented for 80, 60, 40, 20 ms, or were omitted, respectively. There were 80 (4 × 4 × 5) different combinations of target, prime and prime duration (20, 40, 60, 80, and 100 ms) that were presented 5 times each leading to 400 trials altogether.

## Results

### Priming experiment

Trials with reaction times (RTs) deviating more than 2.5 standard deviations from the mean RT of each participant and each experimental condition (2.1%) were excluded.

Mean RTs for correct responses and error rates for each combination of the within-subjects factors prime duration (20 and 60 ms) and prime congruence (incongruent and congruent) for VGPs and NVGPs (between-subjects factor group) are given in Table 1. An analysis of variance (ANOVA) on RTs for correct responses with the between-subjects factor group and with the within subject factors prime duration and prime congruence, revealed significant main effects for all single factors: group, *F*_(1, 48)_ = 6.6, *p* = 0.01, η^2^_*p*_ = 0.12, prime duration *F*_(1, 48)_ = 94.2, *p* < 0.001, η^2^_*p*_ = 0.66, and prime congruence, *F*_(1, 48)_ = 249.8, *p* < 0.001, η^2^_*p*_ = 0.84. On average VGPs responded faster than NVGPs (397 vs. 427 ms), participants responded faster after primes that were presented for 20 ms compared to primes that were presented for 60 ms (405 vs. 419 ms), and participants responded faster with congruent primes compared with incongruent primes (395 vs. 429 ms). The interaction prime duration × prime congruence was also significant, *F*_(1, 48)_ = 254.7, *p* < 0.001, η^2^_*p*_ = 0.84. This significant interaction indicates that the congruence effect is larger for primes that were presented 60 ms than for primes that were presented 20 ms (57 vs. 11 ms). The interactions for prime duration × group and prime congruence × group were not significant, *ps* = 0.31. The three-way interaction of prime duration × prime congruence × group was marginally significant, *F*_(1, 48)_ = 3.6, *p* = 0.06, η^2^_*p*_ = 0.97. The marginal three-way interaction was further explored in order to investigate whether the RT congruence effect differed in VGPs and NVGPs for the two prime durations. We analyzed the impact of 20 ms primes and 60 ms primes separately:

An ANOVA on RTs for correct responses after primes that were presented for 20 ms, with the between-subjects factor group and the within subjects factor prime congruence, revealed significant main effects for all single factors: group, *F*_(1, 48)_ = 6.8, *p* = 0.01, η^2^_*p*_ = 0.13 and prime congruence, *F*_(1, 48)_ = 27.4, *p* < 0.001, η^2^_*p*_ = 0.36. The interaction prime congruencex group was also significant, *F*_(1, 48)_ = 5.8, *p* = 0.02, η^2^_*p*_ = 0.11, indicating that the congruence effect for VGPs was larger than for NVGPs (16 vs. 6 ms). Single comparisons show a significant congruence effect for VGPs, *t*_(24)_ = 7.3, *p* < 0.001, and a marginally significant congruence effect for NVGPs *t*_(24)_ = 1.7, *p* = 0.06.

**Figure 2:**
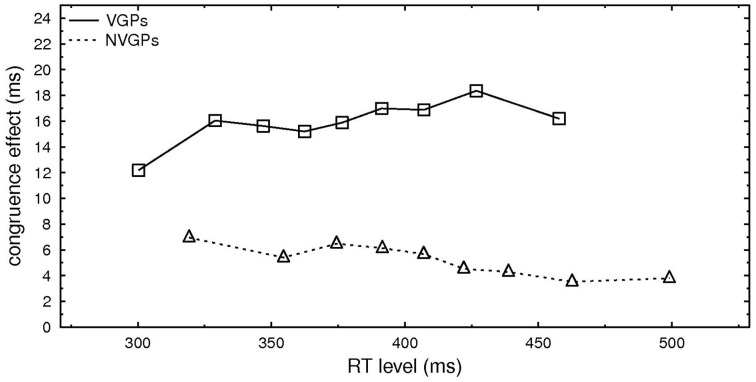
**Congruence effect (in ms) for the nine percentiles (10–90%) depending on the mean RT of each percentile separately for VGPs and NVGPs**.

On average VGPs responded faster than NVGPs. This was also true for primes that were only presented 20 ms (389 vs. 420 ms). To rule out that the difference in the RT levels was responsible for the difference in the congruence effects between VGPs and NVGPs, we examined RT distributions on the basis of percentile values obtained for each participant. For this we rank-ordered RTs per conditions in the 10% fastest, 10–20%,…, 80–90% RTs^3^, computed the average RT per percentile and condition, and computed the congruence effect per percentile by subtracting RT incongruent—RT congruent per percentile. In Figure 2, the congruence effects per percentiles for NVGPs and VPGs are depicted depending on the mean RT of this percentile. The congruence effect for VGPs is rather constant over the percentiles and differences in the size of congruence effects for VGPs and NVGPs occur at similar RT levels.

An ANOVA on RTs for correct responses after primes that were presented for 60 ms, with the between-subjects factor group and the within subjects factor prime congruence, revealed significant main effects for the single factors group, *F*_(1, 48)_ = 6.2, *p* = 0.02, η^2^_*p*_ = 0.12 and prime congruence, *F*_(1, 48)_ = 355.0, *p* < 0.001, η^2^_*p*_ = 0.88. However, the interaction group × congruence was not significant, *ps* > 0.84, indicating that the congruence effect for VGPs did not differ for VGPs and for NVGPs (56 vs. 57 ms).

The overall mean error rate was 4.6%. The same ANOVA on error rates for all responses, revealed significant main effects for the within-subjects factors prime duration *F*_(1, 48)_ = 54.3, *p* < 0.001, η^2^_*p*_ = 0.53, and prime congruence, *F*_(1, 48)_ = 50.1, *p* < 0.001, η^2^_*p*_ = 0.51. Participants made more errors after primes that were presented for 60 ms compared to primes that were presented for 20 ms (6.4 vs. 3.3%) and participants made more errors after incongruent primes compared to congruent primes (7.1 vs. 2.6%). The interaction prime duration × prime congruence was also significant, *F*_(1, 48)_ = 30.6, *p* < 0.001, η^2^_*p*_ = 0.39. The congruence effect was larger for primes that were presented 60 ms than for primes that were presented 20 ms (7.7 vs. 1.3%). The between-subjects factor group was not significant, as well as the interactions for prime duration × group, prime congruence × group, and prime duration × prime congruence × group, *ps* = 0.36.

### Prime visibility

**Figure 3:**
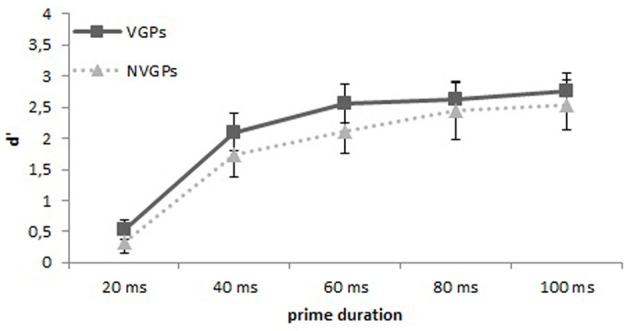
**Mean *d*′s and 95% between-subjects confidence intervals separately for each prime duration for VGPs and NVGPs**.

To assess prime visibility, we computed the signal detection measure *d*′. Prime pictures requiring the response “small” (animal) were treated as signal, whereas prime pictures requiring the response “large” (animal) were considered as noise. Hits and false alarms proportion of zero or one were corrected according to the log-linear rule (Goodman, 1970; cited according to Hautus, 1995) if participants had 0% hits or 100% false alarms. Prime visibility was above chance level for each group and prime duration: Separately for the different prime durations the discrimination performance for VGPs was for primes that were presented 20 ms *d*′ = 0.53, *t*_(24)_ = 6.80, *p* < 0.001, for primes that were presented 40 ms *d*′ = 2.09, *t*_(24)_ = 14.43, *p* < 0.001, primes that were presented 60 ms *d*′ = 2.55, *t*_(24)_ = 16.98, *p* < 0.001, for primes that were presented 80 ms *d*′ = 2.63, *t*_(24)_ = 18.47, *p* < 0.001, and for primes that were presented 100 ms *d*′ = 2.75, *t*_(24)_ = 19.07, *p* < 0.001 (see Figure 3). For NVGPs the discrimination performance for the different prime durations was for primes that were presented 20 ms *d*′ = 0.33, *t*_(24)_ = 3.95, *p* < 0.001, for primes that were presented 40 ms *d*′ = 1.74, *t*_(24)_ = 10.05, *p* < 0.001, for primes that were presented 60 ms *d*′ = 2.11, *t*_(24)_ = 12.28, *p* < 0.001, for primes that were presented 80 ms *d*′ = 2.44, *t*_(24)_ = 11.10, *p* < 0.001, and for primes that were presented 100 ms *d*′ = 2.54, *t*_(24)_ = 13.33, *p* < 0.001 (see Figure 3).

An ANOVA with the between-subjects factor group (VGPs and NVGPs) and with the within subject factor prime duration(20, 40, 60, 80, and 100 ms) showed that prime duration had a significant effect on prime visibility, *F*_(4, 192)_ = 179.0, *p* < 0.001, η^2^ = 0.78, whereas the factor group and the interaction group × prime duration were not significant, *ps* > 0.13. However, when only the prime durations are analyzed that were used in the priming experiment, then an ANOVA with the between-subjects factor group (VGPs and NVGPs) and with the within subject factor prime duration (20 and 60 ms) revealed again that prime duration significantly effected prime visibility, *F*_(1, 48)_ = 355.4, *p* < 0.001, η^2^ = 0.88, as well as a significant effect for the factor group, *F*_(1, 48)_ = 4.5, *p* = 0.038, η^2^ = 0.09, providing at least some evidence that VGPs could discriminate the primes better than NVGPs at these two prime durations. The interaction group × prime duration remained insignificant, *ps* = 0.22.

### Correlations of congruence effect, prime visibility, and game experience

Prime pictures that were presented for 20 ms, elicited a larger congruence effect in VGPs than in NVGPs, while prime visibility was higher in VGPs than in NVGPs when only the two prime durations of the priming experiment (20 and 60 ms) are considered. We therefore ran additional analyses in order to investigate how the two results (congruence effect and prime visibility) as well as video game experience are interrelated.

To test whether the congruence effect is related to the prime visibility, a regression analysis as proposed by Draine and Greenwald (1998, see also Greenwald et al., 1995, 1996) was computed. A priming index was calculated for each participant and each prime type, with index = 100 × (RT incongruent—RT congruent) / RT congruent. The indirect effects of individual priming indices were regressed onto the direct effects of individual *d*′ values separately for VGPs and NVGPs. Following this methodological approach a significant slope of the regression would indicate that congruency effects rise with increasing prime visibility, whereas the “regression intercept estimates the magnitude of priming associated with zero perceptibility of the prime” (Greenwald et al., 1996, p. 1700).

The linear regression analysis revealed no significant correlation between *d*′ and the priming index for prime pictures that were presented 20 ms, neither for VGPs with *r* = 0.105, *p* = 0.62, nor for NVGPs with *r* = 0.294, *p* = 0.15. A *post-hoc* Bayes test for correlations using the procedure of Wetzels and Wagenmakers (2012) revealed Bayes factors of 0.17 for VGPs and 0.42 for NVGPs modestly favoring the null hypothesis that the size of the congruency effects and prime visibility are not related to each other. The intercept of the regression was larger than zero for VGPs, intercept = 3.76, *t*_(24)_ = 3.58, *p* < 0.01, indicating that significant congruence effect for 20 ms in VGPS can be expected even at zero visibility in terms of *d*′. In contrast there was no significant intercept for NVGPs, intercept = 0.85, *p* = 0.40.

For prime pictures that were presented 60 ms, the linear regression analysis revealed again no significant correlation between *d*′ and the priming index for prime pictures, neither for VGPs with *r* = 0.100, *p* = 0.63, nor for NVGPs with *r* = 0.160, *p* = 0.45. A *post-hoc* Bayes test for correlations using the procedure of Wetzels and Wagenmakers (2012) revealed Bayes factors of 0.17 for VGPs and 0.21 for NVGPs modestly favoring the null hypothesis for VGPs and NVGPs, indicating again that the size of the congruency effects and prime visibility are not related to each other. Here the intercept could not be interpreted because *d*′ was > 0 for all participants. Thus, the observed congruence effect that is related to zero visibility for primes that were presented 20 ms is reliable for VGPs and independent on individual prime visibility. For primes that were presented 60 ms, the congruence effect is no longer related to null visibility, but it is still independent of individual prime visibility.

Although we found no significant correlation between individual prime visibility and the priming index in VGPs for primes that were presented for 20 ms, it is conceivable that the effect of video game experience on 20 ms prime processing was nonetheless mediated by an enhanced prime visibility. To rule out that differences in prime visibility are the driving force for increased priming effects in VGPs compared to NVPGs we conducted *post-hoc* a partial regression analysis of the variables game experience (the value was set 0 for NVGPs and 1 for VGPs) and priming index considering prime visibility (*d*′) as confounding effect. A multiple regression with game experience and individual prime visibility as predictors and individual priming index as criterion revealed a significant correlation (*r* = 0.389, *p* = 0.021). Importantly, the correlation was also significant, when partialling the factor prime visibility (*r* = 0.286, *p* = 0.046), but not when partialling the factor game (*r* = 2.15, *p* = 0.137). These results indicate that prime visibility as possible mediator cannot completely account for priming effect differences between gamers and non-gamers with 20 ms primes. Instead, data suggest that game experience is directly related to the size of the congruency effects when prime visibility is controlled for.

## Discussion

We compared video game players' (VGPs) and non-video game players' (NVGPs) ability to process shortly presented near threshold-stimuli in a response priming experiment using masked pictures of drawn animals. Reaction times were faster for VGPs than NVGPs. For primes that were presented only 20 ms, VGPs showed a larger prime congruence effect than NVGPs. For primes that were presented 60 ms, both groups showed a substantial congruence effect that did not differ for VGPs and NVGPs. Additionally, an exploratory analysis gives tentative evidence that VGPs detected masked primes that were presented 20 ms as well as 60 ms better than NVGPs (please note, however, that the improved detection performance is restricted to the prime durations applied in the priming experiment and does not generally hold true). Thus, it seems that gaming expertise is accompanied by more efficient stimulus-response translation and tentatively a somewhat improved visual identification of shortly presented visual stimuli. Apparently, video gaming speeds up already the initial neural processing stream, the so called forward sweep, before recurrent neural processing comes in. Cognitive models of stimulus-response translation have assumed two stimulus-response-translation processes, one “response activation” process whereby stimuli automatically activate assigned motor responses, and another “response selection” process, that eventually determines whether activated responses are carried out (Hommel, 1998; Lien and Proctor, 2002). The masked priming effects we studied here are likely mediated by the fast response activation process (Schubert et al., 2008). Hence, gaming expertise conceivably improves the response activation process involved in the present priming task. However, because of the applied correlational design, some inconclusive findings, and due to further differential results between both groups there are a few caveats that have to be considered firstly.

First, VGPs responded on average 30 ms faster than NVGPs, reflecting a general RT advantage of video gaming experts (e.g., Green and Bavelier, 2003; Castel et al., 2005; Bialystok, 2006; Dye et al., 2009; Colzato et al., 2013). In a meta-analysis Dye et al. (2009) showed that VGPs are on average 11% faster than NVGPs whereby no speed accuracy trade-off occurred. In the present experiment VGPs were on average 8% faster than NVGs fitting well to the data of the meta-analysis (Dye et al., 2009). Similarly, overall error rates were numerically increased for VGPs compared to NVGPs and we thus cannot rule out speed accuracy trade-off. Indeed, a *post-hoc* Bayes test (http://pcl.missouri.edu/bf-two-sample) of the main difference of RTs and error rates for VGPs and NVGPs revealed Bayes factors of 0.28 for RTs and 3.56 for errors (Rouder et al., 2009).

More importantly, however, overall faster RTs for VGPs than NVGPs might be the reason for congruence effects differences because prime impact is rather short-lived. For example, Greenwald et al. (1996) showed that masked priming effects decrease rapidly when the SOA between prime and target is longer than 100 ms. A concomitant percentile analysis ruled out this suspicion. It revealed a quite constant congruence effect for VGPs for all percentiles as well as different congruence effects for VGPs and NVGPs at similar RT levels (see Figure 2). Thus the different congruence effects for primes that were presented for 20 ms cannot be explained by the general effect of faster responses for VGPs than for NVGPs.

Second, there is mixed evidence whether or not the larger congruency effect for VGPs compared to NVGPs for primes that were presented 20 ms is related to increased prime visibility of VGPs. In some studies, it has already been demonstrated that congruence effects increase with prime visibility (Greenwald et al., 1996; Kunde et al., 2005). The present experiment somewhat replicates this result because in both groups of participants, primes that were presented for 60 ms elicited larger congruence effects and were better discriminable than primes that were presented for 20 ms. Please note however, that here prime duration is confounded with visibility. Yet, when just considering primes that were presented for 20 and 60 ms, we found a somewhat better prime discrimination performance for VGPs than for NVGPs. To further investigate whether the size of congruence effects for primes that were presented for 20 ms and prime discrimination are related to each other we conducted two additional analyses. On the one hand, regression analyses as well as *post-hoc* Bayes tests for VGPs and NVGPs respectively, suggested that larger congruence effects were not exclusively brought by a higher individual prime visibility. Moreover, a *post-hoc* test indicated that primes that were presented for 60 ms were also better discriminated by VGPs than by NVGPs, although the amount of congruence effects was equal in both groups. On the other hand, a partial correlation analysis controlling for indirect mediation through prime visibility showed that the direct effect of video gaming expertise on the congruence effect for primes that were presented 20 ms was not driven fully by a possible indirect effect of prime visibility. Thus, in the present experiment it seems that video gaming expertise is related to more efficient stimulus-response translation.

Third, regarding prime visibility we found no statistically significant advantage for VGPs compared to NVGPs in the omnibus test of the discrimination task with prime durations ranging from 20 to 100 ms (in 20 ms steps). This is probably because for long prime durations prime detection rates were quite high and might have been insensitive for group differences. However, this explanation is post hoc and has therefore to be treated cautiously. The same holds true for restricting the analysis to the two prime durations used in the priming experiment. When only these two prime durations (20 and 60 ms) were considered in an explanatory analysis results revealed better visual discrimination performance of masked stimuli for VGPs compared to NVGPs. This result is in line with a study of Li et al. (2010) which shows that action video gaming decreases the efficiency of backwards masks. Moreover with masked letter primes it has already been shown that discrimination performance was better for typing experts than for novices (Heinemann et al., 2010). Nevertheless, additional studies may be required to provide more definitive conclusions about possible video game expertise related detection improvements of shortly presented masked primes and to tease out the underlying mechanisms.

Fourth, a problem when comparing experts and novices refers to a placebo-like effect of expectation and motivation (Boot et al., 2011). It might be that based on how participants are recruited, they already expect that their status of expertise shall be investigated and are therefore more eager to show a good performance. In the present study, we selected participants based on their self-reported gaming experience, thus it might be that VGPs were more motivated than NVPGs eventually explaining the observed RT differences and the better prime discriminability of VGPs compared to NVGPs. Yet, for the congruence effect in the masked priming paradigm such biases hardly play a role. During the priming task, participants are hardly aware of the primes that were presented for 20 ms and it is thus not possible to strategically influence the priming effects. This assumption is supported by the finding that primes affected performance largely independent of response times to conscious targets. Our participants also reported no application of reasonable strategies in the priming experiment. (Occasionally methods such as trying to avoid errors or concentrating on probabilities of targets were reported. Yet, this latter strategy is not helpful as primes and targets occurred equally frequent.) Moreover, we avoid contamination through any effects of expectations on prime processing (cf. Kunde et al., 2003) by using only primes that were also presented as clearly visible targets.

Finally, due to the correlational design of our study, the results have to be interpreted very cautiously. For example, it is conceivable that existing differences in cognitive abilities are responsible that people play action video games (cf. Boot et al., 2011; Kristjánsson, 2013). We thus take this study as a first step to demonstrate differences in stimulus-response translation processes for hardly visible stimuli. Of course, to establish unequivocal evidence for causal relation and to elucidate the exact kind of training required to faster process shortly presented stimuli, training studies are necessary.

To conclude, the results of our study are well in line with recent studies that demonstrated that action video game expertise is related to more efficient visual and cognitive processing. This expertise related processing advantage is especially interesting because the effects of expertise, i.e., improvement of executive functions as well as perceptual learning, generalize to new tasks and are not restricted the domain of the original expertise.

In addition, our results are interesting for research on masked priming because our results are well in line with other studies that observed an impact of expertise on prime processing. However, in all other studies expertise-related stimuli such as the own face (Pannese and Hirsch, 2010), the own name (Pfister et al., 2012), pictures of athletic jumps (Güldenpenning et al., 2011), chess configurations (Kiesel et al., 2009), and words in the mother tongue (Schoonbaert et al., 2009) were used to demonstrate expertise-related lager congruence effects. In the present study we used drawn pictures of animals as stimuli that can be assumed to be of equal familiarity for VGPs and NVGPs. Nonetheless VGPs produced larger congruence effects than NVGPs for primes with a very short duration (20 ms), whereas this group difference was eradicated when the prime duration was prolonged (to 60 ms). Thus, expertise related advantages seem restricted to shortly presented primes and they do not need to be restricted to expertise-related stimulus material.

To our knowledge, this is the first study to demonstrate a relation of video gaming and masked prime processing at a conceptual level based on a more efficient stimulus-response translation, extending findings that action video game training increased prime detection in a backward masking setting (Li et al., 2010). Bearing in mind all the connected caveats, it seems nevertheless appropriate to recommend that action video game experience should be considered in masked priming studies. Especially when between-group comparisons of priming effects are reported, action video game experience is an important factor to control for that might account for group differences particularly when investigating small sample sizes.

### Conflict of interest statement

The authors declare that the research was conducted in the absence of any commercial or financial relationships that could be construed as a potential conflict of interest.

## Footnotes

^1^These two participants revealed 36 and 37 points in the SPM, respectively. These scores are about 5 standard deviations below the mean scores and they are clear outliers because the next lowest SPM scores were 47 points in the NVGP group and 48 points in the VGP group, respectively.

^2^We thank Bruno Rossion and Gilles Pourtois who originally commissioned the pictures and Michael J. Tarr for making the pictures available.

^3^The percentile 90–100% contains too many outliers and is thus not considered.
